# Supplementary material for: MetaQTL: a package of new computational methods for the meta-analysis of QTL mapping experiments
Source: BMC Bioinformatics. 2007 Feb 8;8:49. doi: 10.1186/1471-2105-8-49 (PMC1808479; doi:10.1186/1471-2105-8-49)
Supplement: Additional File 7 — MetaQTL Package : jar file and tutorial. This Zip archive contains both the MetaQTL JAR file and the files of the tutorial. [file 1471-2105-8-49-S7.zip › org.inra.metaqtl/doc/index-files/index-20.html]

U-Index


|  |  |  |
| --- | --- | --- |
| **Overview** \| Package \| Class \| Use \| **Tree** \| **Deprecated** \| **Index** \| **Help** | |  |
| **PREV LETTER**   **NEXT LETTER** | **FRAMES**    **NO FRAMES**     **All Classes** |  |


A B C D E F G H I K L M N O P Q R S T U V W X Y Z 

---


## **U**

**unit** - Variable in class org.metaqtl.Qtl.QTLTrait: The unit of the trait. **UNKNOWN** - Static variable in interface org.thalia.bio.IBioCrossTypes: **unload(IBioEntity, Writer)** - Method in class org.thalia.bio.entity.factory.BioEntityFactory: **unload(IBioEntity, OutputStream)** - Method in class org.thalia.bio.entity.factory.BioEntityFactory: **unload(IBioEntity, Writer)** - Method in class org.thalia.bio.entity.factory.MapChartFactory: TODO (non-Javadoc) **unload(IBioEntity, OutputStream)** - Method in class org.thalia.bio.entity.factory.MapChartFactory: TODO (non-Javadoc) **unload(IBioEntity, Writer)** - Method in class org.thalia.bio.entity.factory.MapMakerFactory: **unload(IBioEntity, OutputStream)** - Method in class org.thalia.bio.entity.factory.MapMakerFactory: **unload(IBioEntity, Writer)** - Method in class org.thalia.bio.entity.factory.MapRawFactory: **unload(IBioEntity, OutputStream)** - Method in class org.thalia.bio.entity.factory.MapRawFactory: **unload(IBioEntity, Writer)** - Method in class org.thalia.bio.entity.factory.MapTabFactory: **unload(IBioEntity, OutputStream)** - Method in class org.thalia.bio.entity.factory.MapTabFactory: **unload(IBioEntity, Writer)** - Method in class org.thalia.bio.entity.factory.OntologyTabFactory: **unload(IBioEntity, OutputStream)** - Method in class org.thalia.bio.entity.factory.OntologyTabFactory: **unload(IBioEntity, OutputStream)** - Method in class org.thalia.bio.entity.factory.XmlBioEntityFactory: **unload(IBioEntity, Writer)** - Method in class org.thalia.bio.entity.factory.XmlBioEntityFactory: **update(Reader)** - Static method in class org.metaqtl.factory.MetaGraphParFactory: **updateLeafIdx(int[])** - Method in class org.metaqtl.Tree: **updateLoglikelihood(double[], double[], EMResult)** - Static method in class org.metaqtl.algo.EMAlgorithm: Updates the loglikelihood value. **updateMappingContext(int, int)** - Method in class org.metaqtl.Chromosome: Updates the mapping context of the chromosome. **updateMuVector(double[], double[], EMResult)** - Static method in class org.metaqtl.algo.EMAlgorithm: Updates the vector of mixture components and returns the new values. **updatePiVector(EMResult)** - Static method in class org.metaqtl.algo.EMAlgorithm: Updates the vector of mixture mixings and returns the new values. **updateZMatrix(double[], double[], EMResult)** - Static method in class org.metaqtl.algo.EMAlgorithm: Updates the Z matrix.

---


|  |  |  |
| --- | --- | --- |
| **Overview** \| Package \| Class \| Use \| **Tree** \| **Deprecated** \| **Index** \| **Help** | |  |
| **PREV LETTER**   **NEXT LETTER** | **FRAMES**    **NO FRAMES**     **All Classes** |  |


A B C D E F G H I K L M N O P Q R S T U V W X Y Z 

---
